# Inadvertent accident during balloon-occluded glue injection for facial AVM: rescue procedure using intentional glue injection

**DOI:** 10.1186/s42155-022-00290-6

**Published:** 2022-03-01

**Authors:** Jihoon Hong, Jung Guen Cha

**Affiliations:** Department of Radiology, School of Medicine, Kyungpook National University, 680 Gukchaebosang-ro, Jung-gu, 41944 Daegu, South Korea

**Keywords:** Arteriovenous malformation, Balloon-occluded glue injection, Glue adhesion

## Abstract

**Background:**

Glue embolization during balloon inflation is a novel technique with many advantages. However, the procedure’s major complication is the adhesion of the balloon catheter by glue. Several studies have reported strategies to prevent this. However, no reports have described a rescue method after accidental adhesion occurs.

**Case presentation:**

A 26-year-old male was referred to the department of interventional radiology for sclerotherapy of an aggravating large facial arteriovenous malformation (AVM). We planned a transvenous approach to decrease the velocity of AVM and increase the efficacy of the sclerotherapy treatment. We performed glue embolization of a major draining vein during microballoon inflation. Upon injection of the glue, inadvertent glue reflux occurred, and the microballoon was stuck to the vessel wall. While removing the microballoon catheter, its shaft broke in the guiding catheter. We filled the inner lumen of the guiding catheter with glue and waited for polymerization to fixate the broken microballoon catheter inside the guiding catheter. Fortunately, the stuck microballoon was separated, and two broken pieces of microballoon catheter were removed through femoral vein short sheath.

**Conclusion:**

Intentional glue casting in the outer catheter is very useful when removing anything that is inside the catheter or stuck due to the glue reflux. It can be applied to various similar emergency situations.

## Background

Balloon-occluded glue injection has been adopted for various procedures since being introduced (Hamaguchi et al. 2013, 2015). It can help flow control and avoid the regurgitation of glue. Its chief complication is the adhesion of the balloon catheter to the vessel walls due to glue reflux. Although several studies have reported strategies to prevent the adhesion of the balloon catheter (Kawai et al. 2012; Mine et al. 2020), none have reported a rescue method for balloon adhesion. Herein, we describe a technique we used to extract a stuck microballoon catheter during treatment of a patient for arteriovenous malformation (AVM). The patient provided informed consent and agreed to publish his case details and images.

## Case presentation

**Fig. 1:**
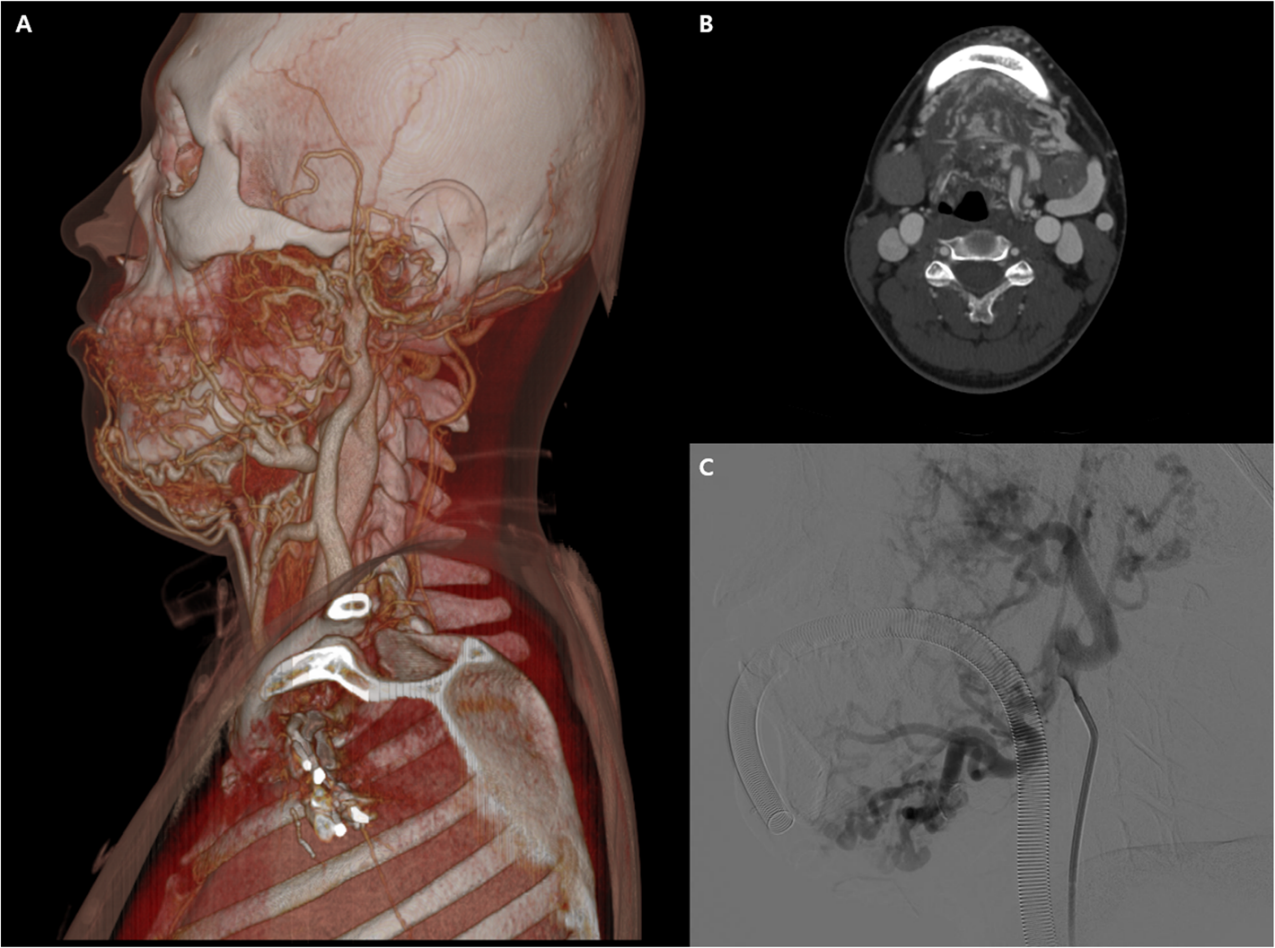
Volume-rendered image (**A**) and axial image (**B**) of CT angiography show huge AVM involving the left side of his face, jaw, tongue, neck, and left ear. **C** Initially performed external carotid artery angiography reveals a very large AVM, which has multiple shunts between the arterioles and venules with dilated fistulae

A 26-year-old male was referred to the department of interventional radiology for sclerotherapy of an aggravating large facial AVM. He was treated with endovascular sclerotherapy in four sessions over 2 years. His initial computed tomography (CT) scan revealed a huge AVM involving the left side of his face, jaw, tongue, neck, and left ear (Fig. 1 A, B). His first external carotid artery (ECA) angiography revealed a very large AVM type IIIb (Fig. 1 C), according to the Cho classification (Cho et al. 2006).

**Fig. 2:**
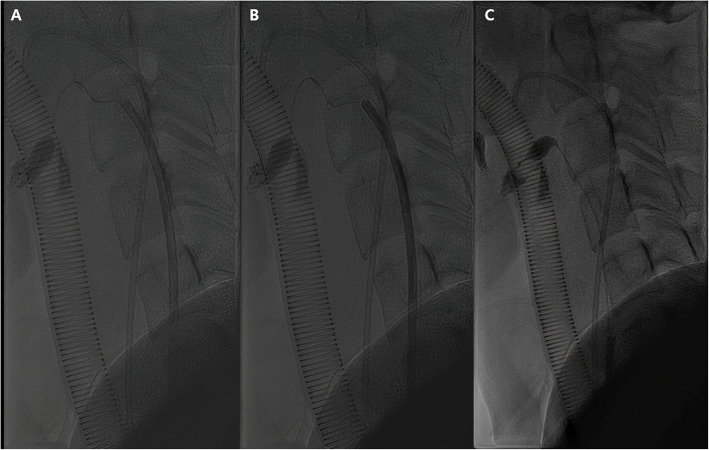
Lateral-view fluoroscopic spot image of the neck. **A** Microballoon catheter stuck to the vessel wall due to the glue cast is seen (**B**) The outer guiding catheter filled with glue is noted. **C** After pulling out the guiding catheter, the stuck microballoon was separated

The procedure was performed in an angio-interventional suite under general anesthesia. Despite four sessions of sclerotherapy through transarterial approach and direct puncture, the AVM peak velocity was still high and some nidus was aggravated. We planned a transvenous approach to decrease the velocity of AVM and increase the efficacy of the sclerotherapy treatment. Access to the arterial and venous system was achieved by ultrasound-guided puncture of the right common femoral artery and right common femoral vein, respectively, using a micropuncture set (Cook Medical, Bloomington, Indiana). According to ECA angiography, we found the major drainage vein, a branch of the internal jugular vein. Then, the major drainage vein was catheterized using a 6-French Envoy guiding catheter (Cordis, Florida, USA) and 5-French headhunter catheter (Cook Medical, Bloomington, Indiana). Using this system, a micro-occlusion balloon (Optimo PB, Tokai medical products, Japan) was coaxially advanced, and venography using the microballoon catheter after inflation showed slower venous flow. Then we planned to use 1 mL of a 1:2 mixture of glue and iodized oil. Upon injection of the glue, inadvertent glue reflux occurred. We had no choice but to wait for the polymerization of the glue to prevent migration to the systemic flow. After a few seconds, we deflated the microballoon and tried to pull it out. However, it was stuck to the vessel wall due to the glue cast (Fig. 2 A). We forcefully pulled it out again. Unfortunately, its shaft broke in the guiding catheter, and the stuck microballoon was still not separated. We filled the inner lumen of the guiding catheter with glue after flushing it with dextrose 5% and waited for polymerization to fixate the broken microballoon catheter inside the guiding catheter (Fig. 2B). Then we pulled out the guiding catheter. Fortunately, the stuck microballoon was separated (Fig. 2 C), and two broken pieces of microballoon catheter were removed though femoral vein short sheath.

## Discussion

AVMs are congenital vascular malformations developed by defects of arterial and venous origins that result in direct communications between vessels of different sizes or primitive reticular networks of dysplastic vessels that have failed to mature into “capillary” vessels called “nidus” (Lee et al. 2013). AVMs have angiographic classification, proposed by Do et al. (Cho et al. 2006; Ko et al. 2019). According to this, our case was AVM type IIIb, which had multiple shunts between the arterioles and venules with dilated fistulae. Direct puncturing of the dilated fistula and injecting ethanol have been proven to be effective, and induced fewer skin complications than transarterial injection (Park et al. 2019). During the last sessions, we performed transarterial and direct puncture alcohol injections. However, these did not work due to the very high velocity of AMVs. Thus, we planned to use a transvenous approach and perform embolization using balloon-occluded glue injection to slow down the velocity and maximize the efficiency of alcohol.

Balloon-occluded glue injection, called the B-glue technique, was introduced by Hamaguchi et al. (Hamaguchi et al. 2013, 2015). This demonstrated many advantages over the flow-dependent technique. It can demonstrate better control of embolus range and less reflux. Moreover, it can also help prevent proximal embolization and distal migration. That said, it has a greater pullback resistance than the flow-dependent technique when reflux occurs due to a larger adhesion area than the usual microcatheter. Furthermore, it is important to remove the microballoon catheter immediately upon reflux detection. In our case, it was performed in venous system and we had no choice but to wait for the polymerization of glue to prevent migration to systemic blood flow.

Several studies have been reported on the prevention of glue reflux and balloon adhesion during the B-glue technique. Mine et al. reported that glue injection via distally advanced microcatheter inserted in the inflated microballoon catheter decreased the risk of glue adhesion to the balloon surface compared with direct injection via a microballoon catheter (Mine et al. 2020). Moreover, Kawai et al. reported that adding ethanol to glue and iodized oil mixture can render microcatheter adhesion negligible. They assumed that rapid completion of N-butyl cyanoacrylate (NBCA) sclerosis by adding ethanol was the cause of the minimal adhesion of NBCA to the catheter (Kawai et al. 2012). However, no reports have been published describing rescue method after adhesion of balloon catheters.

We were able to remove the stuck and broken pieces of the microballoon catheter by filling the guiding catheter with glue to anchor the pieces and pulling them all out together. This technique can be very useful when any object to be removed is inside the catheter or sheath. These conditions can include broken microcatheter within larger catheter and unwillingly deployed detachable or pushable coils that do not entirely come out of the catheter. It is very important to know the internal volume of catheter to prevent overflow of glue while performing this rescue technique.

This technique may also be helpful in removing stuck microcatheters by glue even if they are not broken. In our case, when the microballoon was stuck to the vein wall, we pulled hard on the distal portion of the catheter. However, rather than pulling off, the catheter broke inside the guiding catheter. When we pulled it after filling the guiding catheter with glue, the stuck microballoon fell off. This is probably due to the effect of pulling it from the far proximal of the stuck catheter. It would be more efficient to pull out the catheter adjacent to the adhesion site rather than distal part. In case of unbroken microcatheter stuck by glue, pulling it after advancing outer catheter near the attachment site and making glue casting into outer catheter can be more helpful.

## Conclusions

In conclusion, this intentional glue casting technique is very useful when removing anything inside the catheter and removing things stuck due to the glue reflux. It can be applied to various similar emergency situations.

## Data Availability

Not applicable.

## Abbreviations

AVM: Arteriovenous malformation
CT: Computed tomography
ECA: External carotid artery
NBCA: N-butyl cyanoacrylate
